# Modeling analysis of subthreshold voltage signaling along hippocampal mossy fiber axons

**DOI:** 10.3389/fncel.2022.966636

**Published:** 2022-08-22

**Authors:** Haruyuki Kamiya

**Affiliations:** Department of Neurobiology, Hokkaido University Graduate School of Medicine, Sapporo, Japan

**Keywords:** axon, simulation, voltage, calcium current, mossy fiber

## Abstract

Axons are classically thought of as electrically well isolated from other parts of the neurons due to the shape of a long cable-like structure. In contrast to this classical view on axonal compartmentalization, recent studies revealed that subthreshold depolarization of soma and dendrite passively propagates to the axons for a substantial distance, as demonstrated in some experimentally accessible axons including hippocampal mossy fibers and cortical pyramidal cell axons. Passive propagation of subthreshold dendritic EPSPs to the axons, defined as EPreSPs (excitatory presynaptic potentials), has been demonstrated to affect transmitter release from the axon terminals. To further characterize and explore the functional significance of passive subthreshold voltage signaling along the axons, the model of EPreSPs along hippocampal mossy fibers, proposed by Alle and Geiger, was reconstructed on the NEURON simulator. To test the effect of EPreSPs on action potentials and transmitter release from the axon terminals, additional conductances were incorporated into the previous passive propagation model. These include the axonal sodium, potassium, and leak channels as well as presynaptic calcium channels composed of P/Q-, N-, and R-types, which are reconstructed from the properties of those recorded from mossy fiber boutons experimentally. In this revised model, the preceding subthreshold EPreSPs slightly reduced the action potential-evoked presynaptic calcium currents by a decrease in the amplitude of action potentials due to the slow depolarization. It should be mentioned that EPreSPs by themselves elicited small calcium currents during subthreshold depolarization through these high-voltage activated calcium channels. Since the previous experimental study by simultaneous pre and postsynaptic recordings demonstrated that EPreSPs enhanced action potential-evoked transmitter release from the mossy fiber terminals, it has been suggested that different mechanisms from the enhancement of action potential-evoked presynaptic calcium entry may involve enhanced transmitter release by EPreSP. Small calcium entry by subthreshold EPreSPs may enhance transmitter release from the mossy fiber terminals by acting as high-affinity calcium sensors for enhancing transmitter release. Another form of axonal subthreshold voltage signaling, GABA-EPreSPs elicited by a spillover of GABA from surrounding interneurons, was also explored. Functional consequences of the two modes of axonal subthreshold voltage signaling were discussed with the simulation results.

## Introduction

In most axons, action potentials are generated at the axon initial segment (AIS) and propagate reliably to the distal axon terminals (Bean, 2007; Schmidt-Hieber et al., 2008). In addition to this canonical mode of signaling in the axons, recent studies have revealed that somatodendritic depolarization distributes for a substantial distance by passive propagation due to the cable properties of the axon (Alle and Geiger, 2006; see also Shu et al., 2006), and referred to as excitatory presynaptic potentials (EPreSPs). This non-canonical mode of axonal signaling may add a new modality of analog information processing by axons (Debanne et al., 2011, 2013). Although the functional implication of this non-canonical axonal signaling remains elusive (Ohura and Kamiya, 2016; Trigo, 2019), this mechanism may substantially influence the information transfer through the neuronal network in an activity-dependent manner (Scott et al., 2008; Ohura and Kamiya, 2018a; Kamiya and Debanne, 2020).

The mathematical simulation has been widely used to describe the activities of the axon in the central nervous system, as supplementary to the experimental approach with direct recordings from the axons or the terminals (Engel and Jonas, 2005; Schmidt-Hieber et al., 2008; Diwakar et al., 2009; Ohura and Kamiya, 2018b). The mechanisms underlying the propagation of EPreSPs along the axons were also explored with computer simulations (Alle and Geiger, 2006). They adopted the simple multicompartmental model of the granule cell structure, and successfully reconstructed the passive propagation of EPreSPs quantitatively. However, it was not possible to evaluate the effects on the propagation of action potentials or the subsequent transmitter release from the axon terminals, since the model in their study was incorporated only with passive properties of axonal membranes and not with active conductances. In this study, it was attempted to incorporate the active conductances into the axonal membranes, namely axonal sodium and potassium channels (Engel and Jonas, 2005) as well as the presynaptic calcium channels (Bischofberger et al., 2002) composed of P/Q-type, N-type, and R type (Li et al., 2007) to get insights into the functional consequence of EPreSPs propagation. Using this revised model, action potentials propagating along axons as well as presynaptic calcium current elicited by action potentials were readily calculated, and the consequence of EPreSPs on the axonal propagation and transmitter release were explored.

Here, it was found that the subthreshold EPreSPs slightly reduce the amplitude of the propagating action potentials by the small preceding depolarization. The simulated presynaptic calcium currents during action potentials also reduced the amplitudes. EPreSPs themselves elicited a small calcium current through these calcium channels. Taken together, our simulation data suggested that the facilitated transmitter release observed in the experiments (Alle and Geiger, 2006) attributed to the small calcium entry acting on the high-affinity calcium sensor for synaptic facilitation such as synaptotagmin 7 (Jackman et al., 2016).

In addition, the roles of another form of subthreshold voltage signaling, namely GABA-EPreSPs due to spill-over transmission from the surrounding GABAergic interneurons (Alle and Geiger, 2007) were explored in the mossy fiber model. Since hippocampal mossy fiber axons expressed functional GABA_*A*_-receptors whose activation leads to depolarization due to relatively high intracellular Cl^–^ concentrations in the axonal cytoplasm (Ruiz et al., 2003), activation of axonal GABA_*A*_-receptors potentially modulate the propagating action potentials as well as the subsequent transmitter release. Although the existence of modulatory effects of GABA on the excitability of the mossy fiber axons is shown, the functional consequence of the heterosynaptic actions of GABA has not been explored in detail. In this study, the effects of GABA-EPreSPs were tested on the propagating action potentials as well as presynaptic Ca^2+^ entry during the action potentials reconstructed in the hippocampal mossy fiber model. The simulation demonstrated that GABA-EPreSPs show profound shunting inhibition which reduces the amplitude of axonal action potentials and the presynaptic Ca^2+^ currents evoked by the action potentials, which confirms the notions of the previous experimental study by optically monitoring Ca^2+^ transient at single mossy fiber terminals (Ruiz et al., 2003).

## Materials and methods

### Simulation

Simulations were performed using the NEURON simulation platform version 7.8 for Windows (Hines and Carnevale, 1997). In this study, we adopted the structural model of the granule cell for the reproduction of the simulation of EPreSP propagation (Alle and Geiger, 2006). Briefly, the structure of the granule cell (Geiger et al., 2002) was approximated by dendrites (one main trunk attached with three primaries and nine secondary branches), a soma (diameter, 15 μm), 10 axonal cylinders (diameter, 0.4 μm; length, 150 μm), and 10 *en passant* boutons (diameter, 7 μm) attached with four filopodial extensions (length 20 μm, diameter 0.1 μm). The first axonal cylinder had a proximal diameter of 1 μm and a distal one of 0.4 μm. The hilar collaterals (length 200 μm, diameter 0.2 μm) originated at 50, 100, and 200 μm from the soma, and each carried one large MFB. Figure 2A illustrates the shape of the granule cell model used in this study. The passive electrical properties of the axon including MFBs and filopodial extensions were assumed to be uniform and were the same as for the somatodendritic domain. Specific membrane capacitance Cm 1 μF/cm^2^, specific membrane resistance Rm 60 kΩ cm^2^, and specific intracellular resistivity Ri 70 Ω cm. The time step used in all simulations was 0.05 ms. The resting membrane potential was set to -80 mV.

The models of axonal Na^+^ and K^+^ channels suggested by Engel and Jonas (2005) are based on the data recorded from mossy fiber boutons and reconstructed in our previous study (Ohura and Kamiya, 2018b; Kamiya, 2019b). The model assumed a Hodgkin Huxley-type gating model adapted to channels recorded in mossy fiber terminals, and K^+^ channel inactivation (Geiger and Jonas, 2000) was reconstructed by implementing multiplicatively with parameters of recombinant K_*V*_1.4 channels (Wissmann et al., 2003). The reversal potential of the leak conductance was set to −81 mV to maintain stability. Voltage-gated Na^+^ channels and K^+^ channels were inserted into all compartments of the granule cell model, respectively. The Na^+^ conductance density was set to 50 ms cm^–2^ for the axon and boutons and 10 ms cm^–2^ for the soma. The K^+^ conductance density was set to 36 ms cm^–2^ throughout all parts of the neurons. Action potentials were evoked by injection of depolarizing current into the 9th bouton (0.2 ms, 0.1 or 0.2 nA) or the soma (2 ms, 0.2 nA). The equilibrium potentials for Na^+^ and K^+^ ions were assumed to be +50 and −85 mV, respectively.

The models of presynaptic Ca^2+^ channels of P/Q-type, N-type, and R-type are reconstructed using the kinetic parameters supplied in Table 2 of the paper by Li et al. (2007). The gating models of these presynaptic Ca^2+^ channels assumed six states gating model consisting of five closed states (C0–C4) and a single open state (O) for each subtype. Transitions from C0 to C4 are assumed to be voltage-dependent, while a transition from C4 to O is voltage-independent. To test for the efficacy of presynaptic Ca^2+^ channel activation by propagating action potentials, the Ca^2+^ current at the mossy fiber boutons was calculated.

In addition, spillover transmission from surrounding interneurons (Alle and Geiger, 2007), namely GABA-EPreSPs, was tested for the functional impact on the action potential propagation and the subsequent Ca^2+^ entry to the presynaptic terminals. For the simulation of this form of subthreshold axonal voltage signaling, GABA_*A*_ receptor-mediated conductances were introduced into the axonal membrane in between MFBs at the same density (10 point sources of 0.01 ns distributed along 150 μm of the axon), because the surfaces of MFBs and interleaved in the model are of comparable size. The reversal potential of GABA_*A*_ receptor-mediated currents of −65 mV is taken to be similar to that of somatodendritic GABA_*A*_ receptors of granule cells (Misgeld and Frotscher, 1986). This would correspond to an intracellular chloride concentration of 12 mM for a receptor channel permeable exclusively to chloride ions (Bormann et al., 1987). The reversal potential of −78 mV was chosen to study isolated shunt effects (corresponding to intracellular chloride concentrations of 7 mM), and that of −52 mV (corresponding to 21 mM) was chosen to simulate relatively high presynaptic chloride concentrations as have been observed at the calyx of Held (Price and Trussell, 2006), and neocortical proximal axons (Szabadics et al., 2006). The time course of the simulated GABA_*A*_ receptor-mediated conductance change was chosen such that the resulting current matched the observed spill-over currents.

## Results

### Implementing active conductances into the model of EPreSP propagation

**FIGURE 1:**
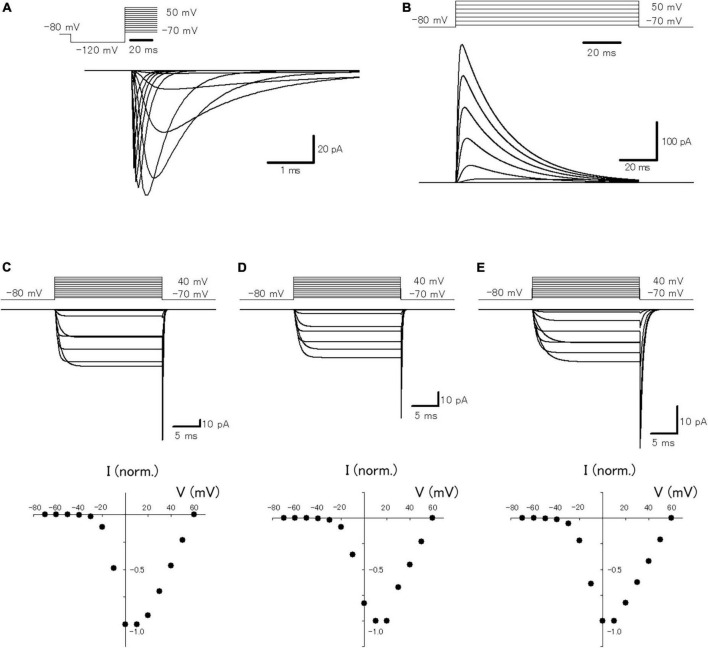
Reconstruction of axonal sodium and potassium current as well as presynaptic calcium current at the hippocampal mossy fiber boutons. **(A)** The voltage-dependency of activation of the simulated axonal sodium current. Traces of calculated sodium current at various test potentials: holding potential at −80 mV for 10 ms, pre-pulse at −120 mV for 30 ms, and test pulses between −70 and +70 mV with 10 mV increments. **(B)** The voltage-dependency of activation of the simulated axonal potassium current. Traces of calculated potassium current by test pulses between −70 and +50 mV with 20 mV increments. The voltage-dependency of activation of the reconstructed P/Q-type **(C)**, N-type **(D)**, and R-type **(E)** components of presynaptic calcium current. Traces of calculated calcium current at various test potentials: holding potential −80 mV, test pulses to between −70 and +40 mV with 10 mV increment, and step back to −80 mV. The lower graphs represent the I–V relationship of the reconstructed P/Q-, N-, and R-type components of presynaptic calcium current.

In a previous study, it was reported that passive propagation of axonal subthreshold voltage signaling was quantitatively reconstructed in a multi-compartment model of hippocampal mossy fibers mimicking the structure of *en passant* axon and the passive properties of cell membranes (Alle and Geiger, 2006). To look for the functional impacts of axonal subthreshold voltage signaling on the spike propagation and the subsequent transmitter release, it was attempted to incorporate active conductances such as the voltage-dependent Na^+^- and K^+^-channels for reconstructing action potential propagation along axons, as well as voltage-dependent Ca^2+^-channels for transmitter release from the axon terminals. For this purpose, we adopted the modified Hodgkin and Huxley-type model that incorporated the experimentally determined gating properties of presynaptic Na^+^ channels (Engel and Jonas, 2005) as well as presynaptic K^+^ channels which shows inactivation (Geiger and Jonas, 2000), reconstructed in Ohura and Kamiya (2018b). To verify that the model reconstitutes the axonal Na^+^-channel properties, simulations of voltage-clamp conditions in the single compartment of a 10 μm sphere were performed (Figure 1A). The calculated Na^+^ current (I_*Na*_) represents similar kinetic properties and voltage-dependency to those reported in the direct recording experiments from the mossy fiber terminals (Engel and Jonas, 2005). For instance, the time constants (τ) of activation and inactivation at −40 mV were 250 μs and 1.75 ms in this simulation, while that determined experimentally was 264 ± 77 μs and 0.95 ± 0.11 ms, respectively. The calculated K^+^ current (I_*K*_) also shows similar gating properties including inactivation during prolonged depolarization (Figure 1B) as experimentally observed by Geiger and Jonas (2000). The time constant of inactivation at 30 mV was 24.5 ms in this simulation, while that determined experimentally was 15.5 ± 0.6 ms. In addition, it was attempted to reconstruct the model of presynaptic Ca^2+^ channels at mossy fiber boutons composed of P/Q-, N-, and R-types. The calculated P/Q-type, N-type, and R-type Ca^2+^ current (I_*Ca*_) represent the kinetics of activation and deactivation as well as voltage-dependency (Figures 1C–E) similar to those observed experimentally (Li et al., 2007). The time constants of activation of P/Q-type, N-type, and R-type I_*Ca*_ at 0 mV were 1.04, 1.07, and 1.62 ms in this simulation, while that determined experimentally was 0.79 ± 0.09 ms, 0.93 ± 0.14 m, and 1.79 ± 0.28 ms, respectively. It should be noted that relatively slower activation, as well as slower deactivation observed as in a tail current, is reconstructed in the model of R-type channels (Figure 1E). The time constants of deactivation of P/Q-type, N-type, and R-type ICa at −80 mV were 0.15, 0.16, and 0.60 ms in this simulation, while that determined experimentally was 0.09 ± 0.01 ms, 0.06 ± 0.01 m, and 0.55 ± 0.08 ms, respectively.

**FIGURE 2:**
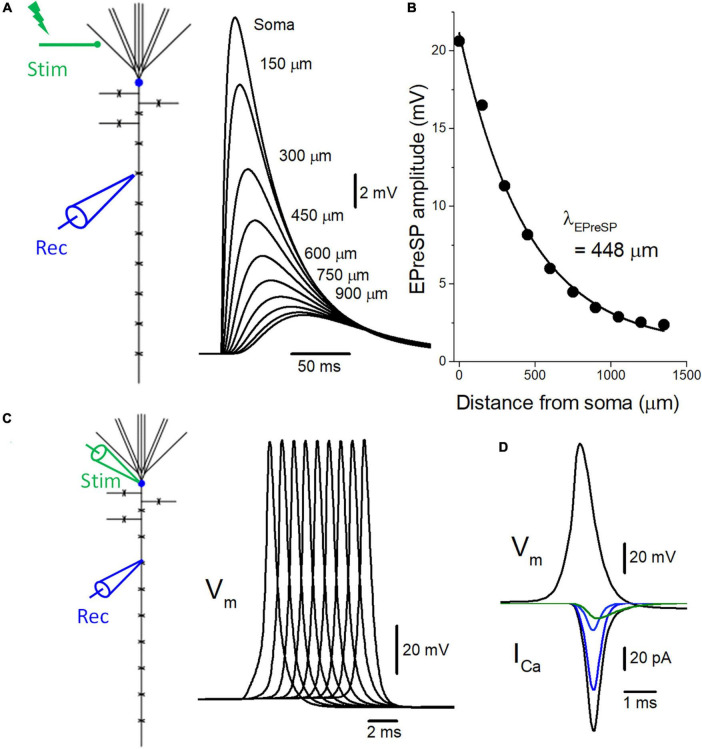
Introduction of active conductance to the passive model of voltage signaling along the mossy fiber axon. **(A)** The multi-compartment model of granule cell was reconstructed according to the previous study (Alle and Geiger, 2006). Somatic depolarization, as well as EPreSPs at the mossy fiber boutons evenly spaced every 150 μm along the main axons elicited by subthreshold dendritic synaptic input, are superimposed. **(B)** The peak amplitudes of EPreSPs are plotted against the distance from the soma. The fitted curve with single exponential decay shows a similar constant λ_*EPreSP*_ = 448 μm to those obtained in the previous experimental study. **(C)** Propagation of action potentials (V_*m*_: upper) throughout *en passant* axon with 9 boutons evenly spaced every 150 μm following stimulation at the soma. **(D)** Simulated propagating action potentials at the mossy fiber bouton (V_*m*_: upper) and presynaptic Ca^2+^ currents (I_*Ca*_: lower) during the action potentials. Total presynaptic calcium current (I_*Ca*_: black) consists of the components representing the sum of P/Q- (dark blue), N- (blue), and R-type (green) calcium channels.

To test for the functional influence of subthreshold EPreSPs on the axonal spike propagation and subsequent transmitter release from the presynaptic terminals, the reconstructed models of voltage-dependent Na^+^, K^+^, and Ca^2+^ channels were implemented into the granule cell model with solely passive membrane properties for the simulation of EPreSPs propagation (Alle and Geiger, 2006) described previously. Consistent with the simulation in the passive model, the subthreshold somatodendritic EPSPs are distributed substantially also into the axon in an active granule cell model (Figure 2A). The calculated EPreSPs at each bouton spaced 150 μm inter-bouton distance gradually decrease the amplitude and the peak time was delayed with the distance from the soma. The peak amplitudes of EPreSPs are plotted against the distance from the soma, and the fitted curve with single exponential decay shows a distance constant λ_*EPreSP*_ = 448 μm (Figure 2B) similar to 430 μm those obtained in the previous experimental study (Alle and Geiger, 2007).

Introducing voltage-dependent Na^+^- and K^+^-channels to the model enabled reconstitute the action potentials propagating along the mossy fiber axons (Alle et al., 2009). Stimulation at the soma generated propagating action potentials reliably toward the distal axons (Figure 2C). In addition, the implementation of presynaptic Ca^2+^ channels enabled the calculation of Ca^2+^-current (I_*Ca*_) elicited by the propagating action potentials (Figure 2D). The time course of I_*Ca*_ nicely reproduced that of experimentally obtained waveforms (Li et al., 2007). Again, the component mediated by R-type showed slower kinetics as shown in the green trace.

### Effect of EPreSPs on propagating action potential and the subsequent presynaptic Ca^2+^ entry

**FIGURE 3:**
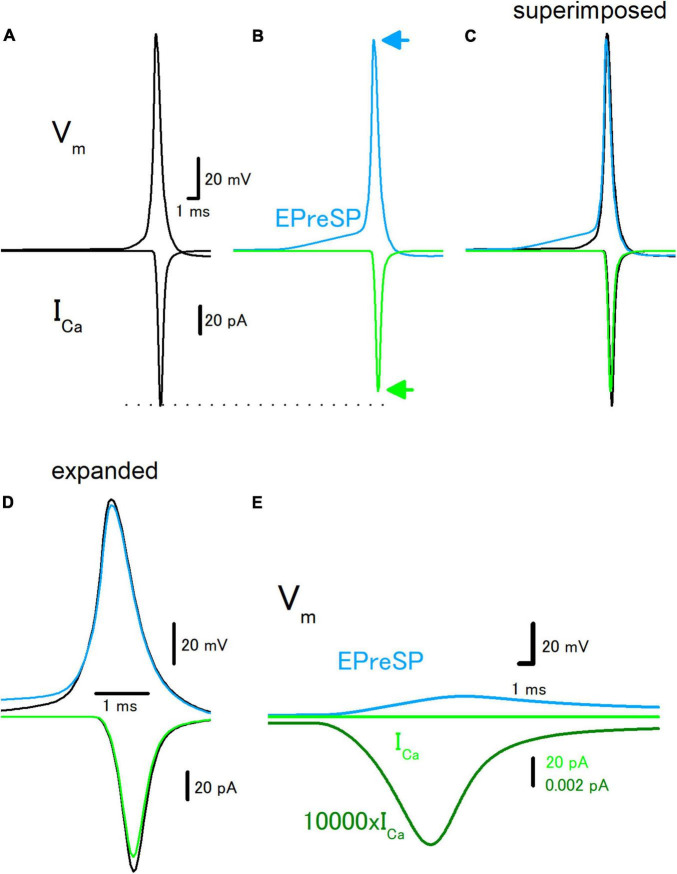
Effects of EPreSP on the propagating action potential and presynaptic calcium current. **(A)** Simulated propagating action potential (V_*m*_: upper) and presynaptic calcium current (I_*Ca*_: lower) of the first mossy fiber bouton of the main trunk in the control condition. **(B)** Propagating action potential (blue) and presynaptic calcium current (green) were calculated with the preceding EPreSPs elicited by stimulation of dendritic input. The synaptic conductance was set to evoke somatic EPSPs of approximately 20 mV. Superimposed **(C)** and time expanded **(D)** traces of propagating action potential and presynaptic calcium current. **(E)** Subthreshold EPreSP (blue)-induced presynaptic calcium current (green) and that of magnified 10,000 times (dark green). Note that the subthreshold EPreSP itself elicited substantial presynaptic calcium current.

Introducing the active conductance to the granule cell model enabled the reconstruction of the propagation of action potentials in the presence and absence of EPreSPs, and passive propagation of dendritic EPSPs distributed to the axon (Alle and Geiger, 2006). Stimulation at the soma elicited action potentials propagating to the axon and I_*Ca*_ was elicited at the axon terminals in the model as described above (Figure 3A). Preceding synaptic input elicited prolonged depolarization of the proximal axon, and the amplitude of action potential decreased by depolarization of EPreSPs (Figure 3B). It should be noted that the peak height of the action potential also slightly decreased, possibly by the inactivation of Na^+^ channels (Figures 3B–D). As expected from the decrease in both the amplitude and the peak height of action potentials, the calculated I_*Ca*_ reduced the peak without affecting the time course significantly (Figures 3B–D). It is also calculated EPreSPs without stimulating the soma, to see whether EPreSPs themselves elicited substantial I_*Ca*_. Although EPreSP alone does not seem to evoke a substantial inward current with the same gain, magnification of the *Y*-axis visualizes the slow inward current through the presynaptic Ca^2+^ channels composed of P/Q-, N-, and R-types (Figure 3E), suggesting that subthreshold depolarization surely activate these high-voltage activated Ca^2+^ channels to some extent. Since the previous experimental studies revealed that EpreSPs enhance the action potential-evoked transmitter release (Alle and Geiger, 2006), reduced presynaptic I_*Ca*_ by preceding EpreSPs may not account for the enhancement of transmitter release. The peak amplitude of I_*Ca*_ was reduced by EPreSPs to 90.3% (from 113 to 102 pA). The charge transfer of I_*Ca*_ was also reduced to 89.4% (from 72.0 to 64.4 fC). Although the mechanisms remain to be elucidated, it was supposed that Ca^2+^ entry by the small I_*Ca*_ during EpreSPs may enhance the transmitter release in a Ca^2+^-dependent manner.

As a molecular basis of the enhanced synaptic transmission by EPreSPs, high-affinity Ca^2+^-sensor molecules are supposed to involve. Among them, synaptotagmin 7 (Jackman and Regehr, 2017) is the leading candidate, for the high affinity for Ca^2+^, slow binding kinetics, and high abundance in many brain regions including hippocampal mossy fibers. It should be noted that the knockout mice of synaptotagmin 7 showed significantly reduced synaptic facilitation at the mossy fiber-CA3 synapses (Jackman et al., 2016).

### Effect of GABA-EPreSPs on propagating action potential and the presynaptic Ca^2+^ entry

**FIGURE 4:**
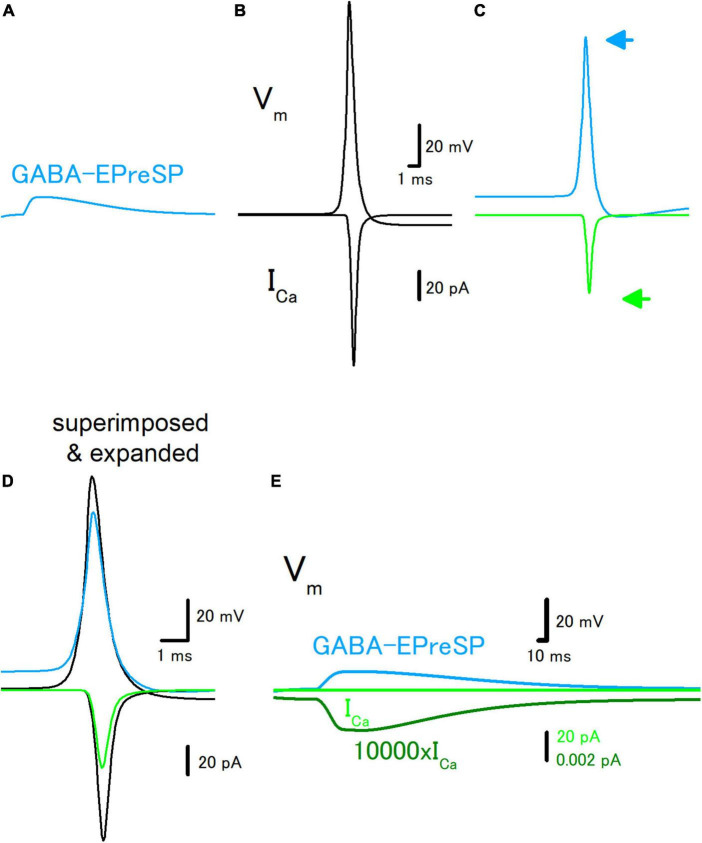
Effects of GABA-EPreSP on the propagating action potential and presynaptic calcium current. **(A)** Simulated depolarizing GABA-EPreSPs (blue) elicited by the activating presynaptic GABA_*A*_ receptors assuming the reversal potentials of −52 mV positive than the resting membrane potentials. **(B)** Simulated propagating action potential (V_*m*_: upper) and presynaptic calcium current (I_*Ca*_: lower) of the first mossy fiber bouton of the main trunk in the control condition. **(C)** Propagating action potential (blue) and presynaptic calcium current (green) were calculated with the preceding GABA-EPreSPs elicited by stimulation of presynaptic GABA_*A*_ receptors. The synaptic conductance was set to evoke somatic EPSPs of approximately 20 mV. **(D)** Superimposed and time expanded traces of propagating action potential and presynaptic calcium current. **(E)** Subthreshold GABA-EPreSP (blue)-induced presynaptic calcium current (green) that magnified 10,000 times (dark green).

To further explore the roles of subthreshold voltage signaling in the modulation of axonal functions, then we examined the effect of GABA-EPreSPs (Alle and Geiger, 2007), a subthreshold depolarization of axonal membranes caused by the spill-over transmission from the surrounding GABAergic synapses. Mossy fibers express functional GABA_*A*_ receptors on the axonal membrane and activation of the GABA_*A*_ receptors enhances the excitability of the mossy fiber axons (Ruiz et al., 2003). The GABA-EPreSPs were calculated by injecting slow synaptic conductance on the assumption that the equilibrium potential of Cl^–^ as −52 mV assumes a higher Cl^–^ concentration in the axon terminals (Price and Trussell, 2006), and displayed depolarizing GABA-EPreSPs (Figure 4A) those similar to obtained experimentally (Alle and Geiger, 2007). Action potentials and the subsequent I_*Ca*_ (Figure 4B) were pronouncedly suppressed by the preceding GABA-EPreSPs (Figure 4C), as expected from shunting effects (Cattaert and El Manira, 1999) expected to influence GABAergic transmission more significantly. When the traces with or without GABA-EPreSPs were superimposed, the time course was not affected much but only the peak amplitudes were suppressed, consistent with the contribution of shunting inhibition (Figure 4D). The peak amplitude of I_*Ca*_ was reduced by GABA-EPreSPs to 51.7% (from 107 to 55.3 pA). The charge transfer of I_*Ca*_ was also reduced to 53.0% (from 69.0 to 36.6 fC). As in the case of EPreSPs, small presynaptic I_*Ca*_ can be visualized with higher magnification, although it is almost undetectable with the original magnification (Figure 4E).

## Discussion

In this study, numerical simulations using a realistic model of hippocampal mossy fiber were performed to illustrate the roles of subthreshold voltage signaling on the propagating action potentials as well as transmitter release processes. EPreSPs, passively propagated dendritic EPSPs into the axon and reduced the amplitudes of action potentials presynaptic calcium current (I_*Ca*_) by the slow depolarization. GABA-EPreSPs, another form of subthreshold depolarization of axons by spill-over transmission from surrounding inhibitory neurons, also reduced the amplitudes of action potentials presynaptic I_*Ca*_ by strong shunting of GABA_*A*_ receptor activation.

### Reconstruction of action potentials and presynaptic Ca^2+^ current in the granule cell model

In the previous study (Alle and Geiger, 2006), the authors experimentally demonstrated that dendritic EPSPs distribute to the axonal membranes for a substantial distance and were termed as EPreSPs. They also demonstrated that numerical simulation in the reconstructed granule cell model with passive membrane properties reconstructs the EPreSPs distribution quantitatively. However, the model has not included the active conductance components, and therefore hardly evaluates the effects on the propagating action potentials and subsequent presynaptic Ca^2+^ entry. So far, the model of Na^+^ and K^+^ channels on the mossy fiber axons was reconstructed according to the paper by Engel and Jonas (2005). In addition, the realistic models of Ca^2+^ channels were reconstructed according to the kinetic parameters obtained by direct recordings from the mossy fiber terminals (Li et al., 2007). By introducing these models of voltage-dependent channels, simulations of EPreSPs, as well as propagating action potentials and subsequent presynaptic Ca^2+^ current, were enabled. It may offer a unique opportunity to evaluate the modulation of axonal functions by subthreshold voltage signaling. Taking advantage of the modeling approach, this study aims at determining the impacts of subthreshold voltage signaling, namely EPreSPs and GABA-EPreSPs, on the propagating action potentials and the presynaptic Ca^2+^ currents. It should be noted that a sufficiently realistic simulation enables extrapolation of the biophysical mechanisms for modulation of the presynaptic Ca^2+^ dynamics underlying synaptic modulation and plasticity.

### Reduction of presynaptic Ca^2+^ current by preceding EPreSPs

To illuminate the functional impacts of subthreshold voltage signaling on the axonal functions, the effects of preceding EPreSPs, passive propagated somatodendritic EPSPs into the axon, were explored with the model of mossy fibers incorporated with the active conductances. Our simulation demonstrated that EPreSPs slightly reduced the amplitudes of the propagating action potentials, possibly by reflecting the inactivation of voltage-dependent Na^+^ channels. Due to this reduction of the amplitude of action potentials, the presynaptic Ca^2+^ current elicited by the propagating action potentials was suppressed by the preceding EPreSPs. Taking account of the steep non-linear dependency of Ca^2+^ entry on the transmitter release (Wu and Saggau, 1994; Zucker and Regehr, 2002), this may result in the substantial reduction of transmitter release from the mossy fiber terminals.

In the previous experimental study, it was found that the preceding EPreSPs enhanced synaptic transmission (Alle and Geiger, 2006). Although the exact mechanism underlying this synaptic enhancement by the preceding EPreSPs remains elusive, the results of the present simulation study suggested that the Ca^2+^ current elicited by the propagating action potentials is predicted to decrease due to the voltage dependency of the gating properties of the presynaptic Na^+^, K^+^, and Ca^2+^ channels. This indicates that some additional mechanisms are involved to counteract the predicted decrease in Ca^2+^ entry. One candidate mechanism would be the facilitation of presynaptic Ca^2+^ current observed in the Calyx of Held axon terminals (Cuttle et al., 1998) by Ca^2+^-dependent mechanisms (Tsujimoto et al., 2002; Mochida et al., 2008; Leal et al., 2012). Since our Ca^2+^-channel model on the hippocampal mossy fiber terminals does not undergo facilitation when tested with a paired-pulse protocol of 50 ms interval of the short depolarizing pulse to 0 mV for 1 ms (not shown), it is speculated that Ca^2+^-dependent facilitation of presynaptic Ca^2+^ channels contributes to counteract suppression of Ca^2+^ entry during propagating action potentials by the preceding EPreSPs. Alternatively, slow depolarization by EPreSPs may inactivate axonal K^+^-channels (Geiger and Jonas, 2000; Alle et al., 2011) and prolong the duration of action potentials to enhance the presynaptic Ca^2+^ entry. In this view, it should be noted that our previous experimental study demonstrated that presynaptic Ca^2+^ current during action potentials at hippocampal mossy fiber terminals facilitate by the afterdepolarization (ADP), a slow subthreshold depolarization following the action potentials lasting for tens of milliseconds, to a small extent (Ohura and Kamiya, 2018b; Kamiya, 2019a). The contribution of the subthreshold-active K_*v*_7 potassium channels (Martinello et al., 2015) or the kainate-type glutamate receptors (Schmitz et al., 2001) on the mossy fibers was also supposed for regulation by subthreshold voltage signaling. Identification of ionic as well as biophysical mechanisms for this facilitation of presynaptic Ca^2+^ current would be the issue to be clarified in future investigations.

### Reduction of presynaptic Ca^2+^ current by preceding GABA-EPreSPs

For a more comprehensive understanding of subthreshold voltage signaling in axons, a similar test has been adopted for the GABA-EPreSPs due to heterosynaptic activation of axonal GABA_*A*_ receptors due to spill-over transmission from the surrounding inhibitory synapses (Alle and Geiger, 2007). Hippocampal mossy fiber express GABA_*A*_ receptors (Bergersen et al., 2003), and activation of these receptors enhances the excitability of mossy fiber axons (Ruiz et al., 2003). Possible higher Cl^–^ concentration in axon than in somatodendritic compartment (Price and Trussell, 2006) resulting in depolarization by activation of presynaptic GABA_*A*_ receptors (Szabadics et al., 2006). Simulation in this study revealed that the preceding GABA-EPreSPs suppressed the action potential amplitude and the Ca^2+^ current. It is worth noting that the effect is more prominent for GABA-EpreSPs than EPreSPs, as expected from shunting (Segev, 1990; Graham and Redman, 1994) due to the increased Cl^–^ conductance by activation of GABA_*A*_ receptors (Staley and Mody, 1992). Although spill-over transmission by GABA enhances the excitability of the axons, it may suppress synaptic transmission at the mossy fiber-CA3 synapse at each synaptic contact by a prominent shunting effect by GABA. This notion was also supported by the measurement of presynaptic Ca^2+^ transients from a single mossy fiber terminal revealed that activation of presynaptic GABA_*A*_ receptors suppressed the presynaptic Ca^2+^ transients (Ruiz et al., 2003). This form of modulation may be drawn as a close analogy of presynaptic inhibition at the primary afferent synapse (Nicoll and Alger, 1979) in that depolarizing action of presynaptic GABA_*A*_ receptors leads to the suppression of transmitter release from the axon terminals.

In this study, a series of numerical simulations using a sufficiently realistic model of hippocampal mossy fiber was performed to illustrate the functional consequence of subthreshold voltage signaling along the axon. EPreSPs by passive propagation of dendritic EPSPs into the proximal portions of the axons are expected to reduce the presynaptic Ca^2+^ entry during action potentials, while the subthreshold depolarization may elicit a small Ca^2+^ current by itself. It was speculated that the enhanced synaptic transmission by EPreSPs observed in the previous experimental study (Alle and Geiger, 2006) may be explained by assuming Ca^2+^-dependent facilitation of the transmitter release process. Since it was demonstrated that the hippocampal mossy fibers express abundant synaptotagmin 7 which critically be involved in the facilitation of a wide dynamic range at this synapse (Jackman et al., 2016), the contribution of this high-affinity Ca^2+^-sensor molecule, as well as facilitation of P/Q-type Ca^2+^ channels as well as action potential broadening of action potential by accumulated inactivation of K^+^ channels (Geiger and Jonas, 2000) are supposed to be involved. Despite the unveiled detailed mechanisms, this non-canonical mode of voltage-signaling axon may add extreme complexity to the information transfer at the hippocampal mossy fiber synapse.

## Data availability statement

All datasets generated for this study are included in the article.

## Author contributions

HK performed the simulation, analyzed the data, and wrote the manuscript.
